# Interplay between nitrogen fertilizer and biological nitrogen fixation in soybean: implications on seed yield and biomass allocation

**DOI:** 10.1038/s41598-018-35672-1

**Published:** 2018-11-30

**Authors:** Santiago Tamagno, Victor O. Sadras, Jason W. Haegele, Paul R. Armstrong, Ignacio A. Ciampitti

**Affiliations:** 1 Department of Agronomy, Kansas State University, 2004 Throckmorton Plant Science Center, Manhattan, Kansas 66506 USA; 2 South Australian Research and Development Institute, Adelaide, Australia; 3 WinField United, Land O’Lakes., Mahomet, Illinois 61853 USA; 4 USDA-ARS, Manhattan, Kansas 66502 USA

## Abstract

Legumes rely on soil mineral nitrogen (N) and biological N fixation (BNF). The interplay between these two sources is biologically interesting and agronomically relevant as the crop can accommodate the cost of BNF by five non-mutually exclusive mechanisms, whereby BNF: reduces shoot growth and seed yield, or maintains shoot growth and seed yield by enhanced photosynthesis, or reduced root:shoot ratio, or maintains shoot growth but reduces seed yield by reducing the fraction of shoot biomass allocated to seed (harvest index), or reducing concentration of oil and protein in seed. We explore the impact of N application on the seasonal dynamics of BNF, and its consequences for seed yield with emphasis on growth and shoot allocation mechanisms. Trials were established in 23 locations across the US Midwest under four N conditions. Fertilizer reduced the peak of BNF up to 16% in applications at the full flowering stage. Seed yield declined 13 kg ha^−1^ per % increase in RAU_R6_. Harvest index accounted for the decline in seed yield with increasing BNF. This indicates the cost of BNF was met by a relative change in dry matter allocation against the energetically rich seed, and in favor of energetically cheaper vegetative tissue.

## Introduction

Globally, soybean [*Glycine max* (L.) Merr.] is a major source of protein and oil. In the US, soybean is grown in a range of latitudes and environments representing 29% of the national crop acreage^1^. Significant breeding effort during the last century sought to improve seed yield and maintain seed protein^2^. One of the challenges to further improve soybean seed yield is the high demand of nitrogen (N) in comparison to cereals and oilseed crops^3,4^. Legumes rely on two sources of N: mineral N from soil and biological fixation (BNF); the proportion from each source varies with environmental and soil conditions including temperature^5^, soil moisture^6,7^, soil pH^8^, mineral soil N^9^, strain^10^ and crop genotype^7,11^. In soybean, BNF is greater in genotypes with longer reproductive periods reflected in maturity group (MG)^12^.

Biological N fixation requires plant’s reduced carbon (C) and energy, as reviewed by Kaschuk *et al*.^13^. For soybean, BNF requires 6-7 g C g^−1^ N in comparison to 4 g C g^−1^ N for assimilation of mineral N; integrated over the growing season the difference in cost is substantial, with potential implications for seed yield and seed protein or oil concentrations. The cost of BNF can be partially compensated by increase in photosynthesis of plants associated with rhizobia^13^ or shifts in allocation of biomass. For instance, nodulated roots accumulated less biomass compared with plants growing with high soil N supply^14^ and lower biomass partitioning to seeds associated with increasing BNF^7^. Thus, the crop can accommodate the cost of BNF by five non-mutually exclusive mechanisms, whereby N fixation: (a) reduces shoot growth and seed yield, or maintains shoot growth and seed yield by (b) enhanced photosynthesis^13^, or (c) reduced root:shoot ratio^15^, or maintains shoot growth but reduces seed yield by (d) reducing seed oil and protein concentration in seed, or (e) the fraction of shoot biomass allocated to seed (i.e., harvest index; HI).

Further, there is an agronomic interest on the role of mineral N to support high seed yield^16,17^ and avoid protein dilution^18,19^. A recent review of Mourtzinis *et al*.^20^ concluded that N fertilization has a small and inconsistent effect on soybean seed yield. This conclusion is, however, largely based on generic trials where coarse fertilization regimes were established to shift the contribution of mineral N and BNF. In contrast, a full-N treatment devised with a careful experimental protocol to ensure an ample N supply during the entire crop season increased soybean seed yield by 11% in relation to unfertilized controls, with a range from no effect for stressful environments (ca. 2500 kg ha^−1^) but increases of 900 kg ha^−1^ in high potential environments (ca. 6000 kg ha^−1^)^16^.

The goal of this study was to investigate the effect of fertilizer N application on BNF and its implications for soybean seed yield and seed protein concentration. We tested the hypothesis that the cost of N fixation is mediated by reduced biomass, reduced allocation to seed captured in the HI^21^, and reduced concentration of protein and oil in seed. Quantification of these effects will provide insights of BNF impact on crop C and N economy, and will contribute to explain the apparent inconsistency in soybean seed yield responses to N fertilization.

## Results

### Effect of N fertilizer on N fixation

**Figure 1:**
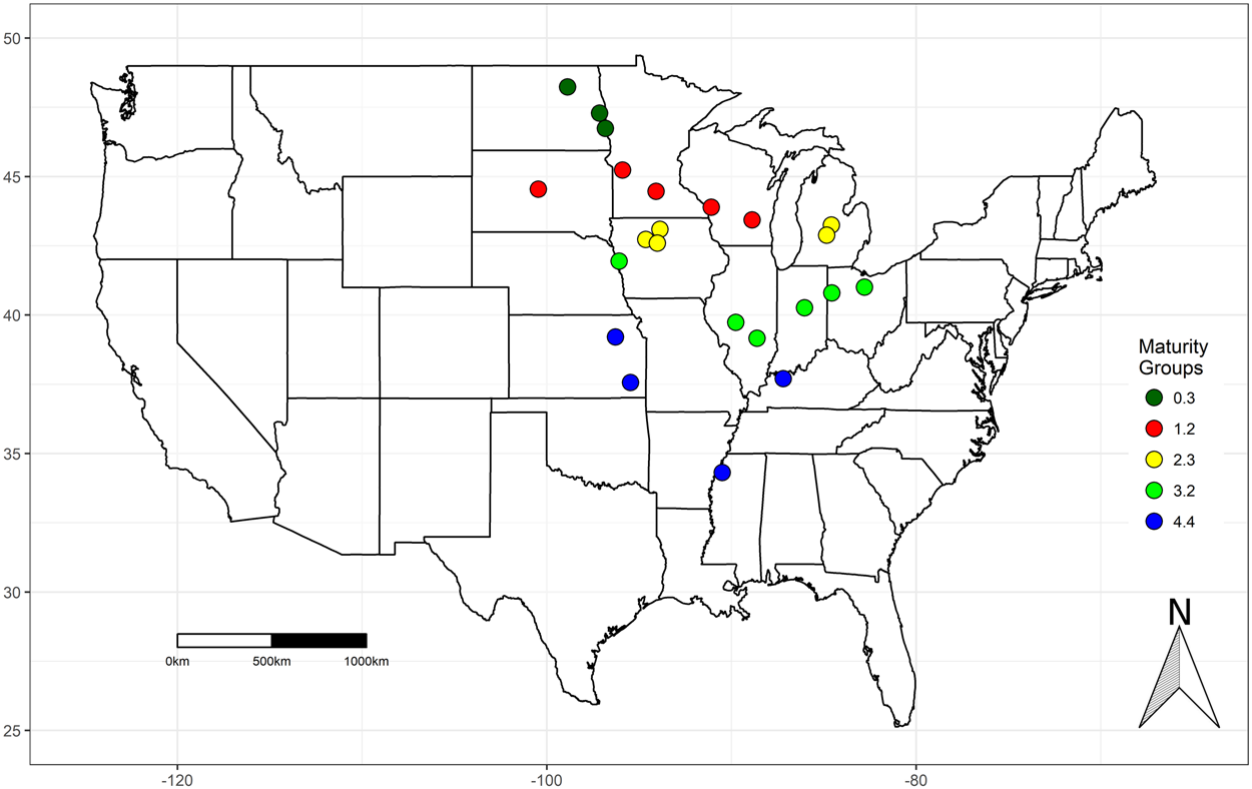
Map of the United States referencing all the experimental locations. Colors represent different soybean maturity groups.

**Figure 2:**
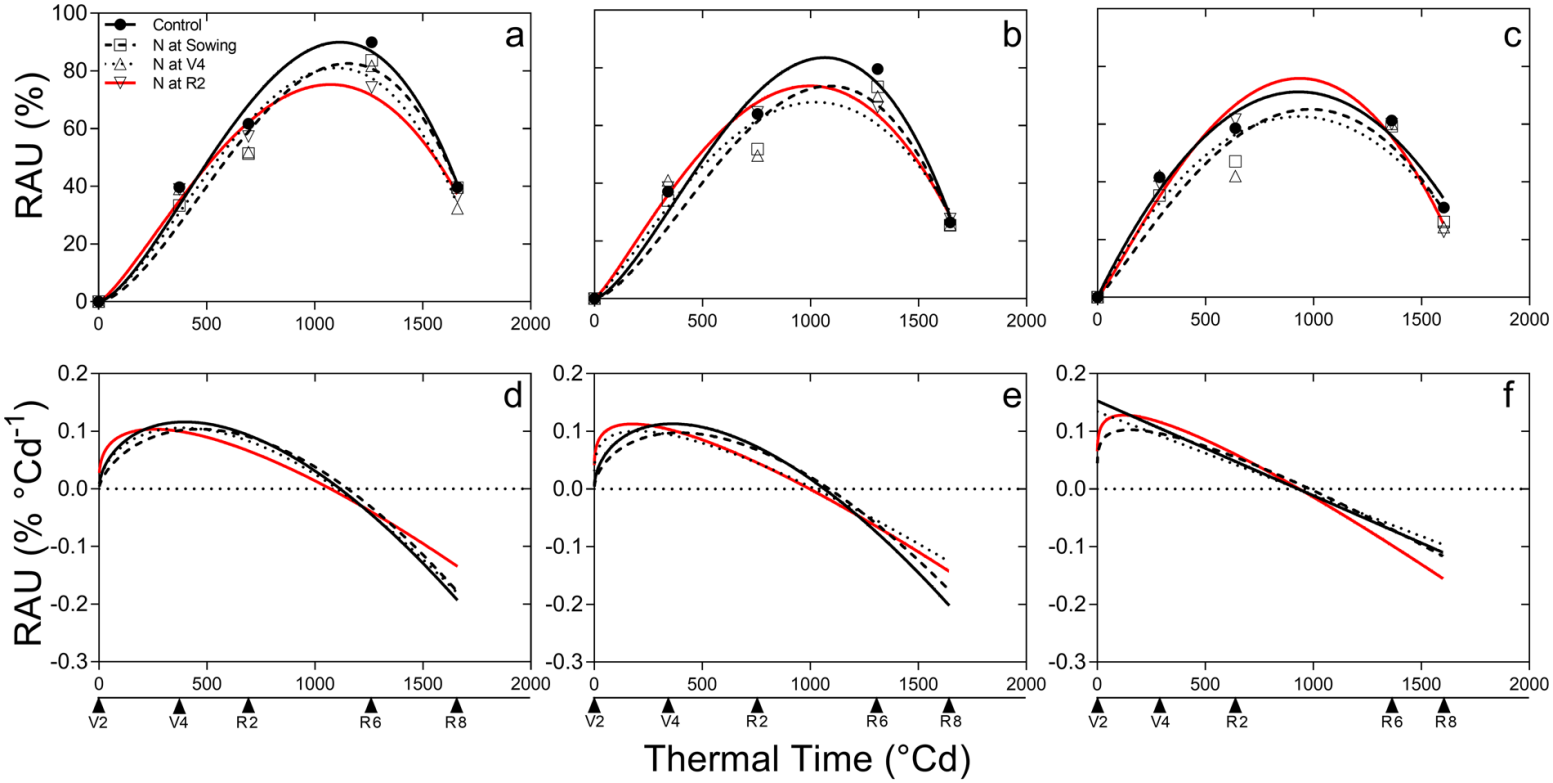
Seasonal changes in the proportion of the relative abundance of ureides (RAU) and RAU rate for the high (**a**,**d**), medium (**b**,**e**), and low (**c**,**f**) BNF groups for the control (black full line and black circles), N at sowing (broken line and empty squares), N at V4 (dotted line and empty triangles), and N at R2 (red full line and empty inverted triangles). Each point in V4 (fourth-leaf), R2 (full flowering), R6 (full seed), and R8 (full maturity) represents the average from the locations of each group.

Data for this study were collected from 23 different locations across the US Midwest during the 2016 growing season (Table 1; Fig. 1). Table 2 shows the relative abundance of ureides in R6 (full seed stage; RAU_R6_) for each location and treatment, Fig. 2 illustrates the seasonal dynamics of the relative abundance of ureides (RAU) for crops grouped in high, medium and low BNF, and Table 3 summarizes the parameters of the curves. The fitted model (equation 2) returned R^2^ between 0.62 and 0.87, with *P* < 0.001 in all cases.

**Table 1:** Location, latitude, longitude, variety, maturity group, sowing date, mean temperature (°C), cumulative water supply (rainfall plus irrigation; mm), vapor pressure deficit (VPD; kPa) from sowing to R7, primary tillage, soil texture^77^, clay (%), sand (%), organic matter (OM; g 100 g^−1^), pH and cation exchange capacity (CEC; cmol charge kg^−1^ soil^−1^) for soybean crops in the Midwest of US in 2016.

Location	Latitude (N)	Longitude (W)	Variety	Maturity Group	Sowing date	Temperature	Water supply	VPD	Tillage	Soil Texture	Clay	Sand	OM	pH	CEC
Attica (Ohio)	41° 0′	82° 48′	R2C3323	3.2	25-05	22.1	561	1.08	Strip-Till	Medium	20.5	11.4	2.0	6.5	15.0
Beaver Dam (Wisconsin)	43° 26′	88° 53′	R2C1572	1.2	17-05	19.5	496	0.91	Chisel	Medium	22.5	7.2	4.0	6.7	21.5
Blencoe (Iowa)	41° 56′	96° 5′	R2C3323	3.2	20-05	20.7	527	1.00	Reduced	Fine	47.5	5.5	2.5	7.9	38.5
Britt (Iowa)	43° 5′	93° 52′	R2C2394	2.3	17-05	19.7	501	0.72	Chisel	Medium	15.0	50.0	7.0	7.8	12.1
Clarksdale (Mississippi)	34° 19′	90° 29′	R2C4541	4.4	24-04	25.2	640	1.30	Plow	Medium	14.0	55.0	0.5	5.8	7.5
Devils Lake West (North Dakota)	48° 14′	98° 53′	R2T0313	0.3	12-05	17.1	434	0.78	Chisel	Fine	23.0	40.0	4.0	7.0	19.9
Effingham (Illinois)	39° 9′	88° 37′	R2C3323	3.2	24-05	22.8	580	0.93	Chisel	Medium	15.0	6.0	1.8	5.8	13.2
Fargo (North Dakota)	46° 44′	96° 49′	R2T0313	0.3	25-05	19.7	501	1.04	Chisel	Fine	48.0	5.0	5.0	7.2	37.1
Holloway (Minnesota)	45° 14′	95° 54′	R2C1572	1.2	05-05	18.3	465	0.97	Chisel	Medium	18.5	11.7	5.0	7.2	19.0
Ithaca (Michigan)	43° 15′	84° 35′	R2C2394	2.3	02-06	19.3	490	0.94	Chisel	Medium	14.0	44.8	2.0	6.7	5.2
Le Sueur (Minnesota)	44° 28′	94° 4′	R2C1572	1.2	16-05	19.5	497	0.88	Chisel	Fine	32.0	30.0	7.0	7.5	25.0
Mayville (North Dakota)	47° 17′	97° 8′	R2T0313	0.3	11-05	17.9	454	0.89	Chisel	Medium	48.0	5.0	5.0	7.2	37.1
Owensboro (Kentucky)	37° 42′	87° 11′	R2C4541	4.4	31-05	23.6	600	0.92	Reduced	Medium	13.0	9.0	2.0	6.2	11.6
Pierre (South Dakota)	44° 32′	100° 27′	R2C1572	1.2	13-05	20.4	518	1.61	Strip-Till	Fine	22.0	9.4	3.0	6.4	18.9
Pocahontas (Iowa)	42° 43′	94° 38′	R2C2394	2.3	18-05	19.8	502	0.92	Chisel	Fine	33.0	25.0	7.0	6.5	28.1
Portland (Michigan)	42° 53′	84° 51′	R2C2394	2.3	03-06	20.0	507	1.02	Strip-Till	Fine	33.6	34.2	7.5	6.7	25.0
Springfield (Illinois)	39° 44′	89° 45′	R2C3323	3.2	05-05	22.0	560	0.94	Chisel	Medium	32.0	1.7	4.5	6.0	26.9
Thayer (Kansas)	37° 33′	95° 28′	R2C4541	4.4	10-05	23.0	583	1.04	Strip-Till	Medium	22.0	6.0	3.3	6.4	18.7
Tipton (Indiana)	40° 15′	86° 2′	R2C3323	3.2	27-05	21.5	547	0.93	Plow	Medium	20.0	19.0	2.5	6.5	14.5
Van Wert (Ohio)	40° 48′	84° 34′	R2C3323	3.2	27-05	20.9	531	1.08	Strip-Till	Medium	23.0	7.0	2.5	5.9	19.3
Vincent (Iowa)	42° 35′	94° 1′	R2C2394	2.3	05-05	19.8	504	0.86	Strip-Till	Fine	30.0	31.0	6.0	6.2	25.6
Wamego (Kansas)	39° 12′	96° 17′	R2C4541	4.4	13-05	22.2	564	1.27	Strip-Till	Medium	19.0	15.0	2.5	6.3	16.4
West Salem (Wisconsin)	43° 53′	91° 6′	R2C1572	1.2	02-05	18.4	466	0.87	Chisel	Medium	17.5	13.6	2.5	6.2	15.2

**Table 2:** Seed yield (13.5% moisture), concentration of protein and oil in seed, and relative abundance of ureides at R6 (RAU_R6_) for soybean crops in the Midwest of US.

Group	Site	Control				N at sowing				N at V4				N at R2				Average (n = 12)			
		Seed Yield	Protein	Oil	RAU_R6_	Seed Yield	Protein	Oil	RAU_R6_	Seed Yield	Protein	Oil	RAU_R6_	Seed Yield	Protein	Oil	RAU_R6_	Seed Yield	Protein	Oil	RAU_R6_
		kg ha^–1^	g 100 g^–1^		%	kg ha^–1^	g 100 g^–1^		%	kg ha^–1^	g 100 g^–1^		%	kg ha^–1^	g 100 g^–1^		%	kg ha^–1^	g 100 g^–1^		%
High	Devils Lake	3349	40.2	17.3	93	3445	41.6	16.7	84	3482	40.0	17.7	85	3296	40.8	17.6	73	3393 ± 111	40.6 ± 6	17.3 ± 0.55	84 ± 6
	Vincent	5717	36.3	20.9	91	5997	36.2	20.7	87	6044	34.7	20.7	86	6137	35.7	20.6	89	5974 ± 169	35.7 ± 2	20.7 ± 0.38	88 ± 2
	Pocahontas	5023	37.1	21.1	90	5117	36.9	21.3	88	4964	35.6	20.8	88	5155	36.9	20.3	89	5060 ± 183	36.6 ± 2	20.9 ± 0.38	89 ± 2
	Britt	5348	36.8	20.9	89	5211	35.7	21.3	84	5118	37.1	21.0	83	5234	36.5	20.9	87	5228 ± 212	36.5 ± 2	21.0 ± 0.54	86 ± 2
	Thayer	5069	36.2	21.8	88	4855	37.7	21.3	74	4978	36.8	20.7	68	4773	38.7	21.3	42	4919 ± 285	37.3 ± 12	21.3 ± 0.47	68 ± 12
	Tipton	4614	40.4	20.6	88	4529	39.8	21.1	86	4247	40.9	20.8	80	4466	40.6	20.7	66	4464 ± 109	40.4 ± 6	20.8 ± 0.55	80 ± 6
Medium	Fargo	3344	38.7	20.7	87	3451	39.2	20.2	76	3634	38.1	20.8	68	3459	38.6	20.2	83	3472 ± 81	38.6 ± 6	20.5 ± 0.36	78 ± 6
	Effingham	4405	40.5	22.0	85	4435	39.9	21.7	86	4445	40.2	21.6	84	4478	38.6	22.0	79	4441 ± 343	39.8 ± 6	21.8 ± 0.46	84 ± 6
	Attica	4902	38.3	21.2	84	4902	38.1	21.1	83	5268	40.4	20.7	77	5206	38.7	21.5	80	5070 ± 159	38.9 ± 6	21.1 ± 0.36	81 ± 6
	Owensboro	4731	38.6	21.2	81	4618	36.5	21.1	86	4698	39.4	20.4	75	4471	39.5	21.4	76	4628 ± 137	38.5 ± 5	21.0 ± 0.48	80 ± 5
	Portland	4803	36.8	22.4	81	4921	37.1	21.7	78	5116	40.5	21.3	74	4856	38.9	22.3	56	4924 ± 148	38.3 ± 9	22.0 ± 0.74	72 ± 9
	Mayville	4260	35.1	19.9	80	4314	36.7	20.1	72	4263	36.8	19.7	64	4569	35.1	20.5	67	4352 ± 101	35.9 ± 5	20.1 ± 0.33	70 ± 5
	Clarksdale	5431	38.7	22.1	79	4965	37.4	21.9	82	5229	37.9	21.6	73	5229	37.6	21.9	71	5237 ± 326	37.9 ± 8	21.9 ± 0.68	76 ± 8
	Wamego	5448	37.2	20.2	79	4529	35.9	20.8	49	5298	37.3	20.3	70	5233	36.9	20.5	59	5127 ± 328	36.8 ± 10	20.4 ± 0.47	64 ± 10
	Blencoe	5466	35.6	21.2	77	5304	34.2	21.8	72	5513	35.2	21.4	75	5383	34.4	21.8	77	5416 ± 86	34.9 ± 4	21.5 ± 0.38	75 ± 4
	Le Sueur	4524	36.1	21.4	77	5042	34.5	22.4	77	4625	31.9	23.0	68	4996	36.2	21.6	70	4775 ± 351	34.7 ± 5	22.1 ± 0.69	73 ± 5
	West Salem	5826	37.4	23.0	73	5634	40.1	21.3	66	5667	39.6	22.2	51	5729	39.9	21.9	60	5714 ± 188	39.2 ± 6	22.1 ± 0.47	62 ± 6
	Holloway	5166	35.2	21.7	72	6150	35.7	21.7	65	6060	39.2	19.9	60	6204	36.9	21.2	57	5880 ± 415	36.7 ± 5	21.2 ± 0.61	64 ± 5
Low	Beaver Dam	5132	—	—	72	5304	—	—	62	5094	—	—	55	5307	—	—	72	5209 ± 232	—	—	65 ± 6
	Springfield	4911	38.4	20.2	69	4602	37.7	22.3	63	4784	36.5	22.4	56	4730	36.7	22.5	59	4757 ± 171	37.3 ± 8	21.9 ± 0.73	62 ± 8
	Ithaca	4128	36.9	21.3	60	3976	36	20.7	68	3934	36.0	21.2	73	4566	35.5	20.4	60	4151 ± 230	36.1 ± 8	20.8 ± 0.32	65 ± 8
	Pierre	6201	33.1	23.9	57	6232	33.8	23.0	46	6248	36.1	22.4	63	6326	34.5	23.3	56	6252 ± 259	34.4 ± 6	23.1 ± 0.47	56 ± 6
	Van Wert	4979	40.1	20.6	48	4913	40.4	20.8	57	5282	41.0	20.6	54	5204	41.8	20.2	53	5094 ± 130	40.8 ± 4	20.5 ± 0.46	54 ± 4

Nitrogen treatments are Control, N at sowing, N at V4, and N at R2. Averages for each site include the 95% confidence intervals. Sites are grouped in three classes: high BNF (>75th quartile), medium BNF (25th-75th quartiles), and low BNF (<25th quartile). Within each group, sites are ranked from high to low RAU_R6_.

**Table 3:** Parameters ± standard error of the evolution curves of RAU fitted for crops with high BNF (>75th quartile), medium BNF (25th-75th quartiles), and low BNF (<25th quartile). RAU_max_ is the peak RAU during the growing season (V2 to R7) reached at t_max_, thermal time t_0.5_ when 50% of RAU_max_ is reached, the maximum rate reached at t_m_, and the area under the curve AUC. Syx is the standard deviation of the residuals of the fitted curve, and R^2^ is the coefficient of determination of the fitted curve (all p < 0.001).

Group	Treatment	RAU_max_	*t*_m_	*t*_max_	*t*_0.5_	Maximum Rate	AUC	Syx	R^2^
		%	°Cd	°Cd	°Cd	% °Cd^−1^	% °Cd	%	
High	Control	90 ± 5a^a^	397 ± 74	1117 ± 29a	473	0.116	92586 ± 6648	12.8	0.85
	N at Sowing	83 ± 5ab	461 ± 85	1153 ± 34a	514	0.104	82749 ± 7417	13.8	0.81
	N at V4	81 ± 5ab	388 ± 78	1103 ± 32b	466	0.106	82619 ± 6691	12.2	0.84
	N at R2	75 ± 6b	257 ± 125	1072 ± 45c	422	0.104	82142 ± 9791	15.0	0.75
Medium	Control	84 ± 3a	362 ± 48a	1066 ± 20a	444	0.113	84986 ± 6215	11.1	0.87
	N at Sowing	74 ± 3b	394 ± 63a	1089 ± 26a	464	0.098	75029 ± 7208	12.8	0.78
	N at V4	68 ± 3 cd	199 ± 87b	1023 ± 32ab	365	0.101	75108 ± 6375	13.1	0.75
	N at R2	74 ± 3bd	176 ± 71b	999 ± 27b	349	0.113	80139 ± 7394	11.7	0.82
Low	Control	71 ± 7	0 ± 187	931 ± 58	272	0.153	78102 ± 9439	14.9	0.71
	N at Sowing	65 ± 7	152 ± 164	985 ± 54	337	0.103	68334 ± 7577	14.0	0.68
	N at V4	63 ± 8	0 ± 230	933 ± 72	274	0.134	67982 ± 7873	15.6	0.62
	N at R2	76 ± 6	125 ± 114	936 ± 42	313	0.128	77387 ± 8160	12.2	0.80

^a^Different letters represent differences between parameters of the same group according to the AICc comparison.

The RAU_R6_ ranged between 42 to 93% (Table 2) and responded to all three sources of variation: treatment, location and their interaction (P < 0.001). Fertilizer reduced RAU_max_ in all BNF groups, but not where BNF was already low in the control treatment (Table 3). In the high BNF group, RAU_max_ dropped from 90% in controls to 75% in their fertilized counterparts (averaging all N treatments; Table 3); the reduction in RAU was larger when the application of N was delayed from vegetative to reproductive stages. In the medium group, RAU_max_ declined from 84% in controls to 68% in the V4 (fourth-leaf) application and 74% in both sowing and R2 (full flowering) applications, with no clear effect of N application timing.

The reduction in RAU_max_ can be a consequence of a shorter time to peak RAU, a reduced rate or a combination of both effects. Combination of both traits contributed to reduced N fixation in the medium BNF group, as peak RAU and maximum rate were attained earlier in N-fertilized crops. In the low BNF group, N fertilizer reduced RAU rate but time of peak RAU was not affected. Reduction of the area under the curve (AUC) and the time when RAU reached 50% was observed from high to low BNF groups; however, treatments did not affect AUC (Table 3).

Fertilization treatments affected the dynamics of RAU rate. Both the timing of peak rate (*t*_*m*_), and the timing when rate became negative (*t*_*max*_) were delayed from low to high BNF groups (Fig. 2d–f). For the high and medium groups, reproductive N treatment was the most effective reducing both *t*_*m*_ and *t*_*max*_ hence contributing to an overall reduction of RAU_max_.

### Effect of N fixation on seed yield, biomass, harvest index, protein and oil concentration

Seed yield ranged from 3151 to 7175 kg ha^−1^, seed protein concentration from 31.9 to 41.8 g 100 g^–1^, and seed oil concentration from 16.7 to 23.9 g 100 g^−1^ (Table 2). For these traits, ANOVA showed location was a significant source of variation (P < 0.001), with no effect of treatment and its interaction with location (P > 0.05). Total biomass ranged from 6093 to 11376 kg ha^−1^ showing differences only among locations (P < 0.001). Harvest index ranged from 0.37 to 0.56, and was affected by both treatment (P < 0.05) and location (P < 0.001).

**Figure 3:**
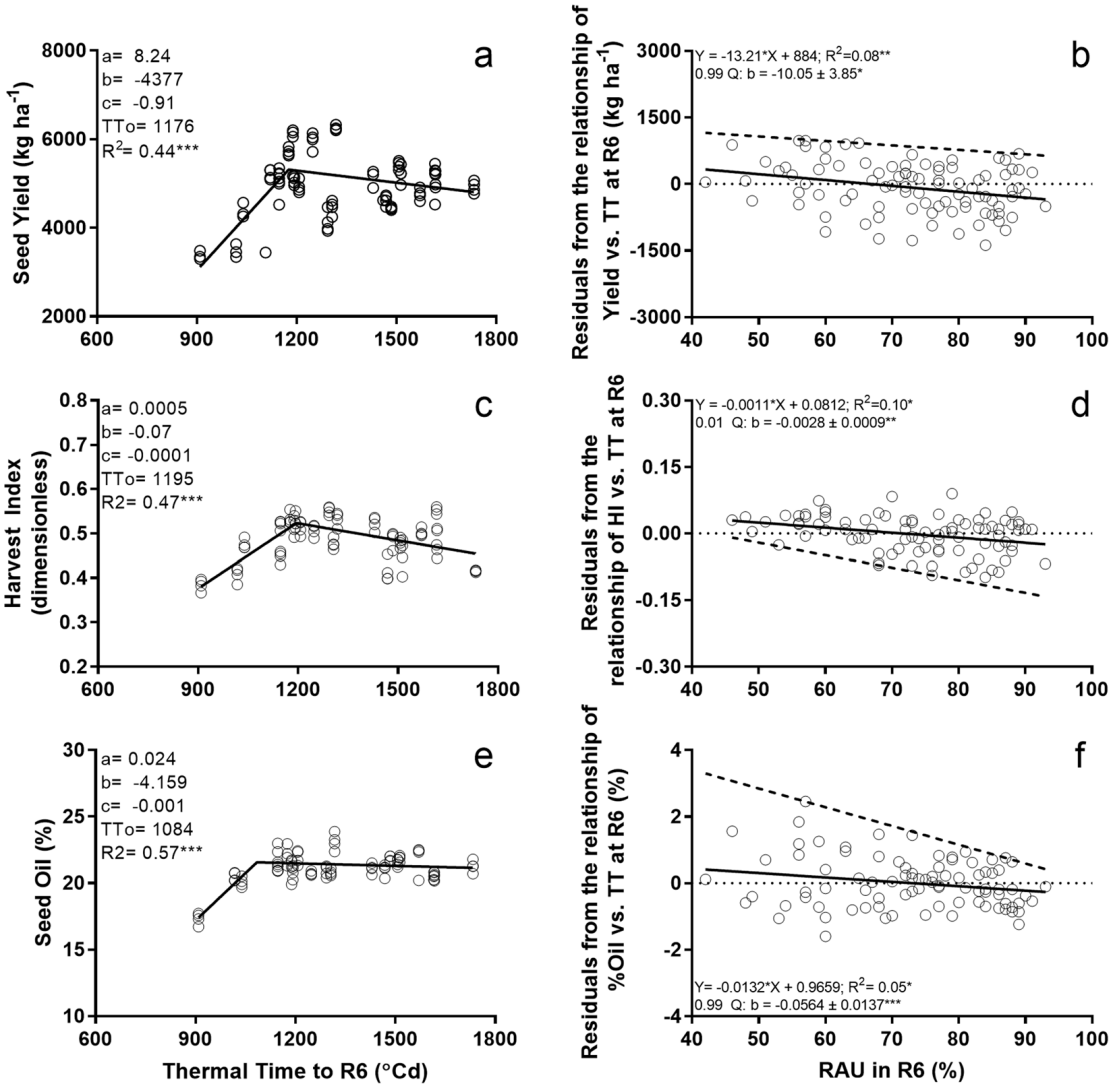
Relationship between seed yield (**a**), harvest index (**c**), seed oil concentration (**e**) and thermal time to R6. In (**a**–**c**), solid lines are bilinear models with fitted parameters a, b, c, TTo (equation 5). Relationship between residuals from relationship in a (**c**), c (**b**), and e (**f**) with RAU at R6. Solid lines are least square regressions, and dashed lines are regressions for the 0.99 (**b**,**f**) and 0.01 (**d**) quantiles. Asterisks indicate significance of the coefficient: three asterisks, P < 0.001; two asterisks, P < 0.01; one asterisk, P < 0.05.

In this experiment, MGs were allocated to locations for agronomic relevance (Table 1; Fig. 1). The dominant effect of location on crop traits is therefore confounded with crop phenology. For instance, the range of thermal time to R6 was 909 to 1733 °Cd. We thus fitted bilinear models to account for the effect of phenology on crop traits (Fig. 3a,c,d), and regressed the residuals against RAU_R6_ (Fig. 3b,d,f; see section on materials and method). Analysis of residuals showed seed yield declined at 13 kg ha^−1^ per % of RAU_R6_ for the whole data set and 10 kg ha^−1^ per % of RAU_R6_ (p < 0.05, Fig. 3b) for top yielding crops (0.99 quartile). Under stressful conditions leading to low HI, the rate of decline in HI with RAU_R6_ computed as the slope of the 0.01 quartile, was 2.5 times larger than the rate for the pooled data (Fig. 3d). Oil seed concentration was negatively associated with RAU_R6_ (p < 0.05; Fig. 3f) and crops with higher oil concentration (0.99 quartile) were more responsive to RAU_R6_. After removing the effects of phenology, biomass and seed protein concentration (not shown) did not relate to RAU_R6_ (both p > 0.05).

**Figure 4:**
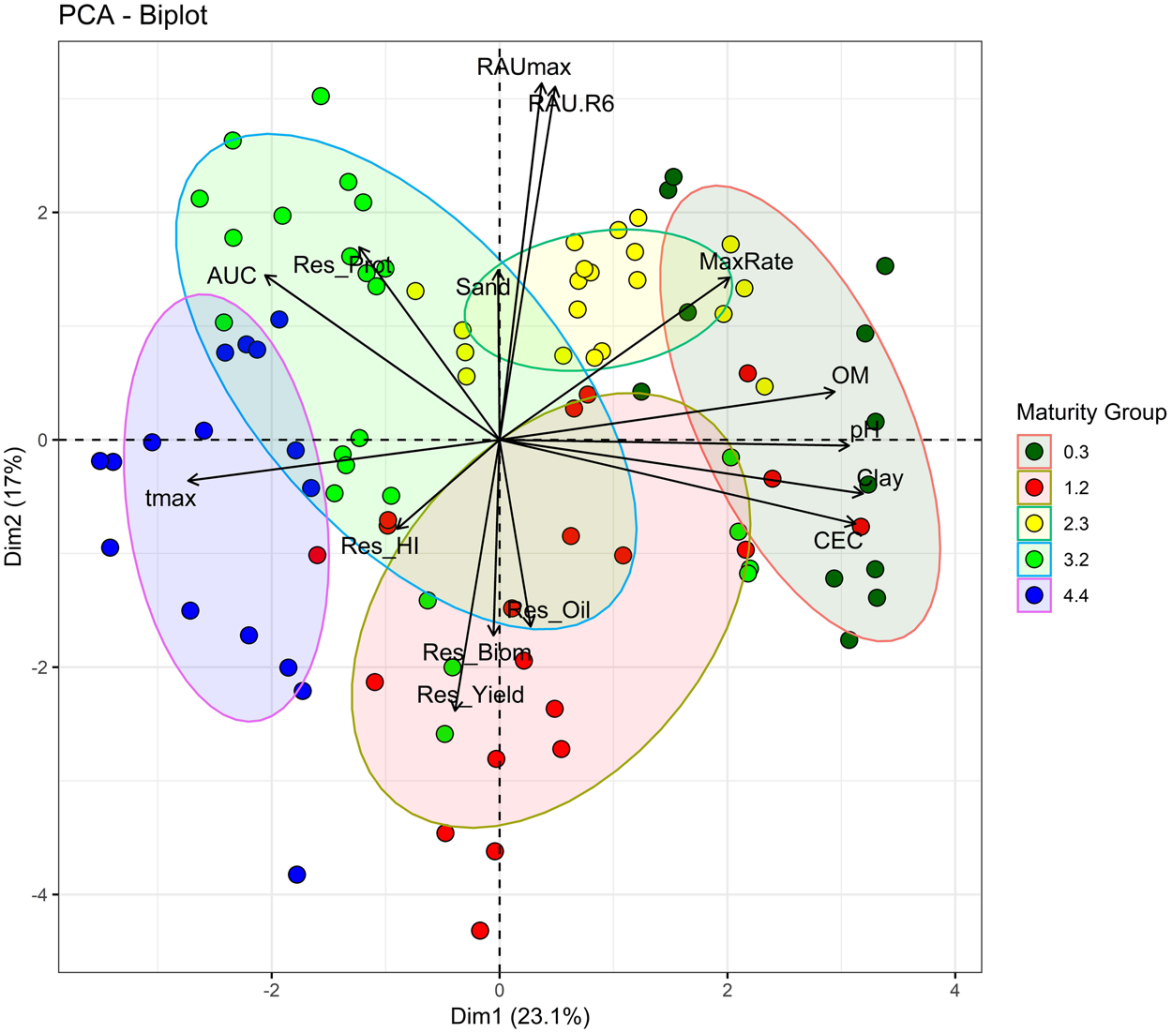
Principal component analysis of crop traits for each combination of treatment and soybean maturity group. Concentration ellipses includes points each maturity group. Traits are maximum rate (MaxRate), residuals from seed protein concentration (Res_Prot), seed yield (Res_Yield), total biomass (Res_Biom), harvest index (Res_HI), oil concentration (Res_Oil), maximum RAU during the growing season (RAU_max_), RAU at R6 (RAU_R6_), relative AUC (AUC), thermal time for RAU_max_ (t_max_), soil organic matter (OM), soil pH, clay, sand, and cation exchange capacity (CEC) from each location.

The association between BNF traits, soil attributes, seed yield, biomass, HI, seed protein and oil concentrations, adjusted by the effects of phenology, were explored using principal component analysis (PCA). Results from the ordination analysis were presented in a bi-plot showing the two first principal components (Fig. 4) where angles between variable vectors denote the level of association among them (i.e., acute angles denote positive associations and obtuse angles negative associations between variables). Pearson correlation analysis complements the associations depicted on the PCA analysis (Supplementary Table S1). Data points from the same MG grouping together showed similarities for the variables of the PCA, reinforcing the dominant influence of phenology previously observed (Fig. 3a,c,e). The MGs, from the shortest to the longest, grouped in the biplot along the first principal component. For instance, data from groups 4.4 and 3.2 were associated with higher AUC when compared to 0.3 MG.

Soil attributes were positively correlated in the first principal component, discriminating the locations with shorter maturity groups and higher values in soil attributes. Interestingly, AUC was negatively correlated with OM, pH, clay and sand percentage, and the RAU_R6_ and rate were positively correlated with the OM and sand (Supplementary Table S1). Seed yield correlated positively with both HI and biomass and negatively with RAU_max_, RAU_R6_, and protein concentration. The AUC correlated positively with RAU_max_ and t_max_ and negatively with the maximum RAU rate.

## Discussion

Biological N fixation in soybean has been quantified at different scales, from field to country^22–24^. The range of RAU in unfertilized controls in our study ranged from 48 to 93%. This compares with an average of 60% of N derived from BNF for the US^23^. In Argentina, BNF in 86 location-years averaged 60% and ranged from 12 to 90%^24^. In Brazil, measurements in 6 environments returned an average of 81% and a range from 69 to 94%^22^. All these studies reflected a similar BNF ceiling around 90%, comparable to the maximum recently reported by Ciampitti and Salvagiotti^25^.

### Nitrogen fertilization reduced BNF and increased seed yield by enhancing C allocation to seed

Phenology was the main source of variation in seed yield and traits associated with BNF clustered with maturity group (Figs 2 and 4). Soybean maturity group influences not only phenology but also growth, and allocation of biomass and nitrogen^26^. Variation in BNF with MG relates to both the duration of the reproductive period when BNF and biomass growth rate peak, and the delay in the exponential phase of BNF^27–29^. In our study, application of N reduced peak and altered dynamics of RAU during the season (Fig. 2), but phenology masked the association between N fixation and seed yield. After removing the dominant effect of phenology, crop yield declined with increasing BNF (Figs 3 and 4). This effect of maturity group has not been considered in previous comprehensive studies^20,30^.

Reduced HI was the primary driver of the reduction in seed yield with increasing RAU_R6_, with an additional weak but significant reduction in oil seed concentration. Low yielding environments showed a steeper decline of HI with increasing RAU_R6_ (Fig. 3d), highlighting the interaction with overall environmental conditions affecting dry matter allocation; stress during reproduction often reduces HI^31,32^.

Changes in plant C allocation in association with BNF have been reported at different levels of organization and time scales. Reduction in BNF associated with phosphorus deficiency altered short-term allocation of C in lupin (*Lupinus luteus*), reducing photosynthesis:respiration ratio, and increasing the ratio between growth respiration and maintenance respiration^33^. Likewise, low magnesium supply altered carbohydrate allocation in soybean, increasing sucrose and starch allocation to leaves that later limited nodule growth^34^. Decreases in biomass allocation in seeds for chickpea (*Cicer arietinum L*.) and pea (*Pisum sativum L*.) were reported with increasing BNF^7,35^. Re-analyzing the data of Sadras *et al*.^7^, where 20 chickpea varieties were grown in 8 environments, HI declined witn BNF at a rate of 0.0022 units per %BNF (Supplementary Fig. S1), in comparison with 0.0011 units per %RAU_R6_ for soybeans in our study; similar to our trial, the decline in HI with BNF was larger for stressed chickpea crops.

Generically, plants require 1 g of glucose to produce either 0.33 g of lipid, 0.40 g of protein, or 0.83 g of carbohydrates^36^. Reducing oil concentration in seed is therefore an energetically effective way to meet the cost of BNF, as found in this study (Fig. 3e). This is in contrast to previous studies where seed protein concentration was reduced and oil concentration did not change in response to BNF^37,38^.

Our findings are in contrast to other studies where N fertilizer reduced BNF but did not increase soybean seed yield^39,40^. For example, Santachiara *et al*.^39^ found no seed yield response in heavily fertilized crops (600 kg N ha^−1^ spread over the season) that reduced BNF to 16% in comparison to 69% in unfertilised controls. However, Santachiara *et al*.^39^ neither report seed yield in equivalent glucose nor changes in protein and oil concentrations. Results from these experiments can be influenced from soil variables influencing BNF activity. In our study, long-term stable soil attributes were included in the analysis. Organic matter was positively correlated with RAU_R6_ and the maximum RAU rate but negatively with the AUC (Supplementary Table S1). The negative association between AUC and soil organic matter might be attributed to the soil N mineralized from N organic fraction during the season^41^. Collino *et al*.^24^ compared average BNF in soybean production systems of Argentina and Brazil, and attributed the lower BNF in the former to better soil fertility.

Of the five putative mechanisms to account for the metabolic cost of BNF, enhanced sink-driven photosynthesis^13^ and reduced root:shoot ratio are the remaining hypotheses to explain the lack of seed yield response with reduced BNF in Santachiara *et al*.^39^. Implicit in the photosynthesis hypothesis is that soybean seed yield is sink-limited; although unlikely, it requires further research. Enhancing photosynthesis by increasing atmospheric carbon dioxide, Ryle *et al*.^42^ reported increases in nodule activity for shadowed white clover (*Trifolium repens* L.) plants but not in their non-stressed counterparts. It is likely that effects of photosynthetic rates on nodule activity depend on reserve carbohydrates^43^, suggesting a link with the differential trends in HI between favourable and poor environments observed in this study. An alternative, less explored mechanism for the maintenance of seed yield in crops relying on N fixation relative to fertilized crops is the reduction in root:shoot ratio; reduced root:shoot ratio is a generic response of plants to high availability of soil N^15^.

### Agronomic and breeding implications

Soybean plays a relevant role in crop rotations^44^ and is a major source of oil and protein worldwide. Improving BNF can be achieved by breeding and selection targeting the plant, the N-fixing bacteria, and better matching plant and bacteria^45–47^. Selection for maintenance of BNF in dry soil has been proposed to improve seed yield of soybean under drought^48,49^. Sinclair *et al*.^49,50^ combined ureide concentration in petioles and acetylene reduction activity to test this proposition. Selected lines were compared with high-yielding commercial cultivars under broad environmental conditions. Two lines were identified that outperformed commercial checks under water deficit, but trade-offs were apparent under high yielding conditions. In this context, the trade-off between BNF and seed yield mediated by HI needs attention. Solving this trade-off needs quantification of the costs (seed yield reduction), agronomic and environmental benefit of BNF. Selection for high biomass partitioning to seed in genotypes growing under low concentration of soil nitrate represents a possible breeding strategy as higher rates of BNF are expressed. In both soybean^51^ and common bean (Phaseolus vulgaris L.)^52^, sensitivity of N fixation to soil mineral N varies with genotype. In alfalfa (Medicago sativa L.), selection for BNF improved plant growth^53^.

## Conclusion

Seasonal characterizations over a wide range of agronomic and environmental conditions revealed that N application reduced maximum RAU at R6, particularly for late applications. At the crop level, soybean met the cost of BNF by a reduction in seed yield mediated by lower HI, particularly in stressful environments, and a secondary contribution of reduced seed oil concentration. The lower-level mechanisms underlying shifts in HI associated with BNF warrant further attention.

## Methods

### Experimental sites and treatments

During the 2016 growing season, soybean N fertilization studies were replicated in 23 sites across the US Midwest in a latitude range from 34°16′ N to 48°14′ N and from 90°29′ W 98°53′ W (Fig. 1, Table 1). Due to the range of latitudes between locations, the length of the growing season differs among sites. Thus, sowing dates and MG were considered following local management practices and recommendations which ranged from 0 to IV due to the large range of latitude in the locations^54^. The seeding rate of 300,000 seeds per hectare, targeted maximum seed yield. Crops were rainfed and received supplementary irrigation to avoid severe water stress (Table 1). On-site weather stations recorded daily temperature, precipitation, and relative humidity; the vapor pressure deficit (VPD, kPa) was estimated using the maximum daily temperature and relative humidity^55^. Soil parameters from every location are presented in Table 1. Data for percentages of clay and sand, organic matter (OM), soil pH and cation exchange capacity (CEC) was extracted from the California Soil Resource Lab (http://casoilresource.lawr.ucdavis.edu, accessed 11 June 2018) using latitude and longitude coordinates from each experiment.

Four treatments were established: an unfertilized control, and 112 kg N ha^−1^ as urea (46-0-0 N-P-K,) at one of three stages: at sowing, at V4 (fourth-leaf), and between R2 (full flowering) to R3 (beginning of pod formation)^56^. The experimental design at each location was a randomized complete block with three replicates. Plot size was 8.4 m long by eight rows at 0.76 m row spacing. The supply of other nutrients was done with N-free fertilizer.

### Phenology, biomass, seed yield, harvest index, seed protein and oil concentration

Plant development stages in relation with calendar time is usually referred as phenology. Phenological stages were recorded during the season following Fehr and Caviness^56^. Shoot biomass samples were collected at the R8 stage (full maturity) from 1.6 linear m and fractioned into stem, leaves, and seeds. The relative proportion of seeds to the total shoot biomass was quantified as the harvest index (HI)^57^. Variations on this ratio can be associated with the influence of the environmental effects on seed yield and biomass production. Seed yield was collected from two-central rows at maturity and adjusted to 13.5 g 100 g^−1^ seed moisture content. Seed samples were collected from harvest for oil and protein determination by near infrared (NIR) spectroscopy using a completely automated Fourier Transform-IR imaging Microscope (Hyperion 3000, Bruker Optics, Ettlingen, Germany) and a sample of >50 seeds. Seed protein and oil concentrations are reported on dry weight basis (Table 2).

### Seasonal dynamics of BNF

Biological N fixation was measured four times, at V4 (fourth leaf), R2 (full flowering)-R3 (beginning of pod formation), R6 (full seed), and R8 (full maturity) using main stem samples. Stems were dried at 65 °C until constant weight and ground to pass through a 2-mm sieve. The BNF percentage was calculated as the relative abundance of ureide-N (RAU) in the main stems using the procedure of Hungria and Araujo^58^. The RAU was calculated as a function of ureides nitrate-N (NO_3_-N) concentration^59^.

1 $${\rm{RAU}}\,( \% )=\frac{{\rm{4}}\times {\rm{Ureide}}\,{\rm{concentration}}}{[({\rm{4}}\times {\rm{Ureide}}\,{\rm{concentration}})+{{\rm{NO}}}_{3}^{-}\,{\rm{concentration}}]}\times {\rm{100}}$$

Time units used to measure the progress of RAU during the season was thermal units (degree-days; °Cd) to account for thermal differences in growing conditions and be independent from the temperature in which different developmental stages occurs. A degree-day is the result from every degree on the daily mean temperature above the base temperature^60^. Thus, cumulative thermal time was calculated as:

2 $${\rm{Thermal}}\,{\rm{Time}}\,(^\circ \mathrm{Cd})={\sum }^{}(\frac{{{\rm{T}}}_{{\rm{\max }}}+{{\rm{T}}}_{{\rm{\min }}}}{2}-{{\rm{T}}}_{{\rm{base}}})$$

where T_max_ and T_min_ is the maximum and minimum daily air temperature (°C), and Tb is the base temperature (8 °C)^61^.

Changes in RAU during the growing season has been described as a sigmoidal pattern with a slow increase early in the season and a maximum attainable between R5 (beginning of seed filling) and R6 (full seed)^62^. However, owing to the large variation in genotypes and growing conditions, RAU at R6 varied widely. To account for this variation, we used RAU at R6 in unfertilized controls to split data into three groups: below the 25^th^ quartile (low BNF), between the 25^th^–75^th^ quartiles (medium BNF), and above the 75^th^ quartile (high BNF). The low BNF comprised five sites with RAU below 72%; the medium BNF included twelve sites with RAU from 72 to 88%, and the high BNF group comprised of six sites with RAU above 88%. For the data combined for each group, the seasonal RAU evolution was described with the beta growth function^63^ with three parameters:

3 $${\rm{RAU}}\,( \% )={{\rm{RAU}}}_{{\rm{\max }}}({\rm{1}}+\frac{{{\rm{t}}}_{{\rm{\max }}}-{\rm{t}}}{{{\rm{t}}}_{{\rm{\max }}}-{{\rm{t}}}_{{\rm{m}}}}){(\frac{{\rm{t}}}{{{\rm{t}}}_{{\rm{m}}}})}^{\frac{{{\rm{t}}}_{{\rm{\max }}}}{{{\rm{t}}}_{{\rm{\max }}}-{{\rm{t}}}_{{\rm{m}}}}}$$

where t is the thermal time from V2 (vegetative leaf), RAU_max_ is the maximum RAU at thermal time t_max_, and t_m_ is the thermal time for maximum RAU growth rate. Biological meaning on parameters allowed us to make inferences on the magnitude of the N treatments on the RAU dynamics by statistical comparisons. Differences between parameters of equation (2) were tested using the Akaike’s Information Criteria (AIC) by performing pairwaise comparisons of individual curves against a global fit. Maximum rate, *t*_*0.5*_, and the AUC where compared using the 95% confidence interval. Both RAU and thermal time were estimated through least squares mean analysis by fitting a mixed model with PROC MIXED procedure^64^ (lsmeans statement) to adjust corrected means to the factors of the model. For this analysis, treatment and locations were considered as fixed factors, and block was nested within location as a random factor. The goodness of fit of the model was assessed with the coefficient of determination (R^2^) and the standard deviation of residuals (Syx)^65^.

Using equation (3), we derived three related traits: the AUC to integrate seasonal N fixation^66^ normalized to the maximum of the data set; *t*_*0.5*_ the thermal time when RAU is 50% of RAU_max;_ and the maximum rate of RAU expressed in changes of % units of RAU per unit of thermal time (°Cd)^63^:

4 $${\rm{Maximum}}\,{\rm{Rate}}\,( \% \,{{\rm{Cd}}}^{-1})=\frac{{{\rm{2t}}}_{{\rm{\max }}}-{{\rm{t}}}_{{\rm{m}}}}{{{\rm{t}}}_{{\rm{\max }}}({{\rm{t}}}_{{\rm{\max }}}-{{\rm{t}}}_{{\rm{m}}})}{(\frac{{{\rm{t}}}_{{\rm{m}}}}{{{\rm{t}}}_{{\rm{\max }}}})}^{\frac{{{\rm{t}}}_{{\rm{m}}}}{{{\rm{t}}}_{{\rm{\max }}}-{{\rm{t}}}_{{\rm{m}}}}}{{\rm{RAU}}}_{{\rm{\max }}}$$

The first derivative of equation (3) with respect to thermal time can be solved to calculate the RAU rate changes across the growing season^63^:

5 $${\rm{RAU}}\,{\rm{Rate}}\,( \% \,{{\rm{Cd}}}^{-1})=\,{\rm{Max}}\,{\rm{.}}\,{\rm{Rate}}\,(\frac{{{\rm{t}}}_{{\rm{\max }}}-t}{{{\rm{t}}}_{{\rm{\max }}}-{{\rm{t}}}_{{\rm{m}}}}){(\frac{{\rm{t}}}{{{\rm{t}}}_{{\rm{m}}}})}^{\frac{{{\rm{t}}}_{{\rm{m}}}}{{{\rm{t}}}_{{\rm{\max }}}-{{\rm{t}}}_{{\rm{m}}}}}$$

Same approach has been utilized to describe other biological process such as N uptake rate in corn^67^ or grain growth rate^68^.

### Analysis of treatment effects and associations between traits

Analysis of variance (ANOVA) was used to investigate effects of treatments on crop traits (seed yield, biomass, HI, RAU_R6,_ seed protein and oil concentration). Sources of variation in ANOVA included N treatment, location, and their interaction as fixed factors, and block as a random effect nested within location; this analysis was implemented by using the R software (version 3.4.0, lme4 package, lmer function)^69,70^.

The effects of BNF on seed yield, biomass, HI, seed protein and oil concentration were analyzed in two steps. First, due to the geographical distribution of the experiments, responses on crop traits are confounded with the different duration of the developmental stages. Thus, effects of phenology were captured with non-linear models:

6a $${\rm{Y}}={{a}}^{\ast }X+b\,{\rm{when}}\,{\rm{X}} < {{\rm{TT}}}_{{\rm{o}}}$$

6b $${\rm{Y}}={{\rm{d}}}^{\ast }{\rm{X}}\,{\rm{when}}\,{\rm{X}}\ge {{\rm{TT}}}_{{\rm{o}}}$$

where Y is the trait, X is the thermal time to R6 (°Cd), and a, b, TT_o_, and d, are parameters. Next, linear regressions and quantile regressions were fitted between residuals of these models and RAU_R6_. This simple approach on the use of residuals allows to netting out^71^ the effect of the phenology on the traits observed when are regressed against RAU_R6_. There are relationships on other parts of the distribution of the response variable that can provide more complete view of the processes studied besides of the mean effect observed. Slopes from quantile regression analysis estimate the changes at the maximum and minimum response that can be missed when other regression methods are used^72^. Thus, regressions for 0.99 and 0.01 quantiles capture the boundaries of the relationships, and were fit in R (quantreg package^73^). The rest of linear and non-linear regression analyses, computation of AUC, and estimation and comparison of parameters from equations (3) and (5) were performed using GraphPad Prism^74^.

Principal component analysis (PCA) was used to analyze general associations between traits allowing the identification of any grouping association within the data set when environmental and crop attributes are analyzed together^75^. Data were classified according to MG, which in turn had a geographical correlation (Table 1). Traits included RAU_max_, t_max_, maximum rate of RAU evolution, seed protein and oil concentration, AUC, residuals from seed yield, biomass, and HI vs thermal time to R6 relationship, and soil attributes (clay, sand, organic matter, pH, and CEC). Principal component analysis was fit using the “FactoMineR” package in R^76^. Pearson correlation coefficients were calculated to complement associations found in the PCA.

## Electronic supplementary material

Supplementary Data
