# Supplementary material for: Interplay between nitrogen fertilizer and biological nitrogen fixation in soybean: implications on seed yield and biomass allocation
Source: Sci Rep. 2018 Nov 30;8:17502. doi: 10.1038/s41598-018-35672-1 (PMC6269449; doi:10.1038/s41598-018-35672-1)
Supplement: Supplementary file 1 — Supplementary Data [file 41598_2018_35672_MOESM1_ESM.docx]

**Interplay between nitrogen fertilizer and biological nitrogen fixation in soybean: implications on seed yield and biomass allocation**

Santiago Tamagno^1*^, Victor O. Sadras^2^, Jason W. Haegele^3^, Paul R. Armstrong^4^ and Ignacio A. Ciampitti^1*^

^1^Department of Agronomy, Kansas State University, 2004 Throckmorton Plant Science Center, Manhattan, Kansas 66506, USA

^2^South Australian Research and Development Institute, Australia

^3^WinField United, Land O’Lakes. Mahomet, Illinois 61853, USA

^4^USDA-ARS, Manhattan, Kansas 66502, USA

*Corresponding authors: [stamagno@ksu.edu](mailto:stamagno@ksu.edu); [ciampitti@ksu.edu](mailto:ciampitti@ksu.edu)

**Supplementary Table S1:** Pearson correlation coefficients (r) between variables of the PCA (Fig. 4). Traits are maximum RAU during the growing season (RAUmax), thermal time for RAUmax (tmax), maximum rate (MaxRate), relative AUC (AUC), RAU at R6 (RAUR6), residuals from seed yield (Res_Yield), total biomass (Res_Biom), harvest index (Res_HI), oil concentration (Res_Oil), seed protein concentration (Res_Prot), soil organic matter (OM), soil pH, cation exchange capacity (CEC), clay, and sand percentage.

|  | RAU_max_ | | t_max_ | | MaxRate | | AUC | | RAU R6 | | Res_Yield | | Res_Biom | | Res_HI | | Res_Oil | | Res_Prot | | OM | | pH | | CEC | | Clay | |
| --- | --- | --- | --- | --- | --- | --- | --- | --- | --- | --- | --- | --- | --- | --- | --- | --- | --- | --- | --- | --- | --- | --- | --- | --- | --- | --- | --- | --- |
|  | | | | | | | | | | | | | | | | | | | | | | | | | | | | |
| t_max_ | -0.04 | ns |  |  |  |  |  |  |  |  |  |  |  |  |  |  |  |  |  |  |  |  |  |  |  |  |  |  |
| MaxRate | 0.32 | ** | -0.83 | *** |  |  |  |  |  |  |  |  |  |  |  |  |  |  |  |  |  |  |  |  |  |  |  |  |
| AUC | 0.50 | *** | 0.46 | *** | -0.18 | * |  |  |  |  |  |  |  |  |  |  |  |  |  |  |  |  |  |  |  |  |  |  |
| RAU_R6_ | 0.76 | *** | 0.11 | ns | 0.02 | ns | 0.31 | ** |  |  |  |  |  |  |  |  |  |  |  |  |  |  |  |  |  |  |  |  |
| Res_Yield | -0.18 | * | 0.12 | ns | -0.15 | ns | 0.10 | ns | -0.29 | ** |  |  |  |  |  |  |  |  |  |  |  |  |  |  |  |  |  |  |
| Res_Biom | -0.16 | ns | 0.07 | ns | -0.03 | ns | -0.13 | ns | -0.14 | ns | 0.60 | *** |  |  |  |  |  |  |  |  |  |  |  |  |  |  |  |  |
| Res_HI | -0.13 | ns | -0.04 | ns | 0.04 | ns | 0.18 | ns | -0.32 | ** | 0.40 | *** | -0.29 | ** |  |  |  |  |  |  |  |  |  |  |  |  |  |  |
| Res_Oil | -0.08 | ns | -0.01 | ns | -0.10 | ns | -0.17 | ns | -0.21 | * | 0.11 | ns | 0.12 | ns | -0.09 | ns |  |  |  |  |  |  |  |  |  |  |  |  |
| Res_Prot | 0.07 | ns | -0.03 | ns | 0.14 | ns | 0.15 | ns | 0.01 | ns | -0.28 | ** | -0.39 | *** | -0.04 | ns | -0.33 | ns |  |  |  |  |  |  |  |  |  |  |
| OM | 0.10 | ns | -0.41 | *** | 0.30 | ** | -0.32 | ** | 0.22 | * | 0.04 | ns | -0.05 | ns | -0.04 | ns | -0.08 | ns | -0.18 | ns |  |  |  |  |  |  |  |  |
| pH | 0.05 | ns | -0.39 | *** | 0.26 | * | -0.43 | *** | 0.16 | ns | -0.01 | ns | 0.07 | ns | -0.16 | ns | -0.06 | ns | -0.35 | *** | 0.50 | *** |  |  |  |  |  |  |
| CEC | 0.03 | ns | -0.27 | ** | 0.08 | ns | -0.16 | ns | 0.05 | ns | 0.07 | ns | -0.10 | ns | -0.17 | ns | 0.09 | ns | -0.25 | * | 0.49 | *** | 0.46 | *** |  |  |  |  |
| Clay | 0.05 | ns | -0.29 | ** | 0.13 | ns | -0.20 | * | 0.07 | ns | -0.06 | ns | -0.12 | ns | -0.18 | * | 0.09 | ns | -0.25 | * | 0.45 | *** | 0.47 | *** | 0.97 | *** |  |  |
| Sand | 0.21 | * | -0.03 | ns | 0.25 | * | -0.21 | * | 0.31 | ** | -0.16 | ns | 0.22 | * | -0.05 | ns | -0.13 | ns | -0.11 | ns | 0.19 | * | 0.13 | ns | -0.44 | *** | -0.33 | ** |
|  | | | | | | | | | | | | | | | | | | | | | | | | | | | | |

* p ≤ 0.10; ** p ≤ 0.01; *** p ≤ 0.001; ns: p > 0.10.


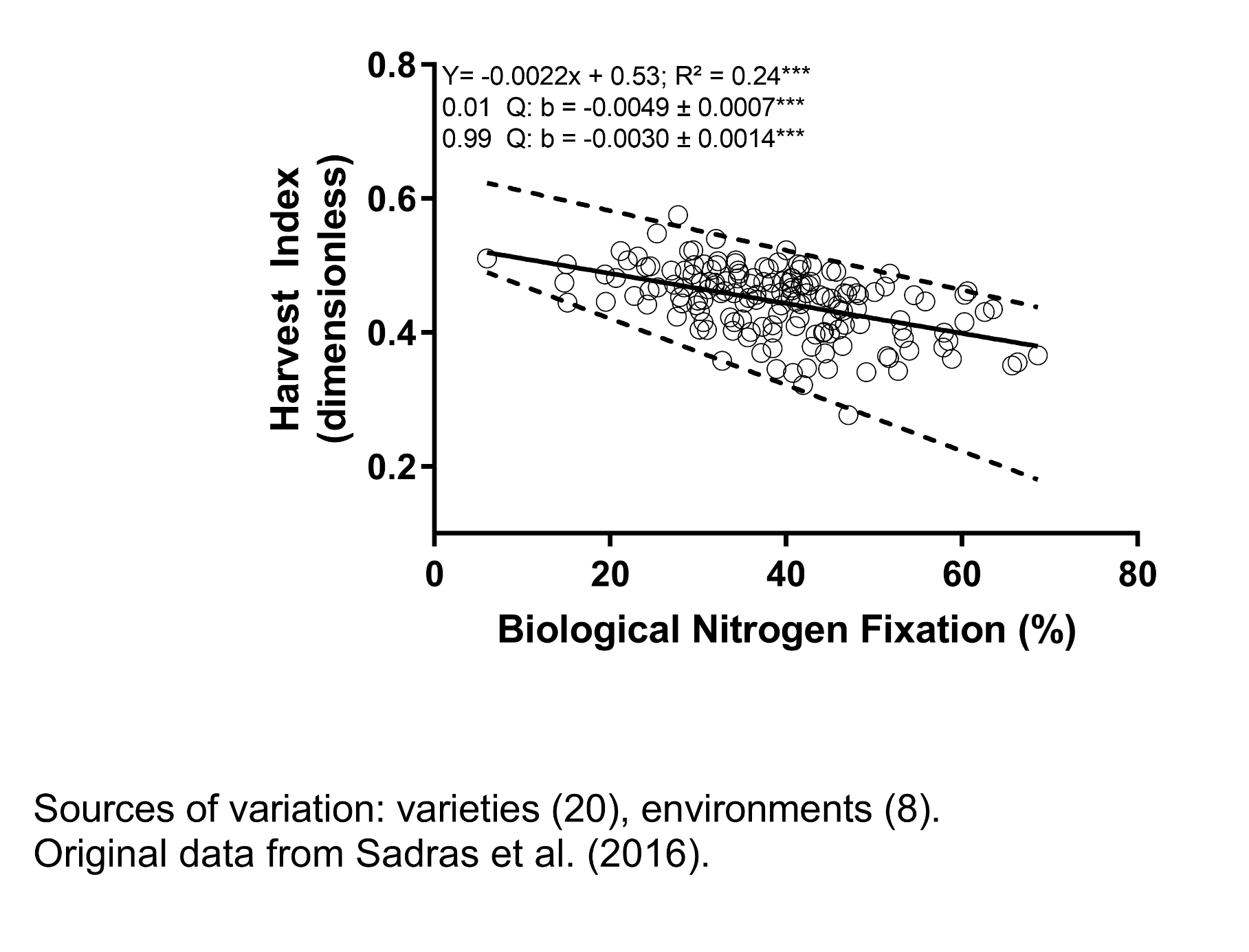


**Supplementary Figure S1:** Relationship between harvest index and biological nitrogen fixation for 20 chickpea (Cicer arietinum L.) varieties grown in eight different environments. Solid line represent the least square regression, and dashed lines are regressions for the 0.99 and 0.01 quantiles. Asterisks indicate significance of the coefficient: three asterisks, P<0.001. Original data from Sadras et al.^8^
